# High-sensitivity tunneling magneto-resistive micro-gyroscope with immunity to external magnetic interference

**DOI:** 10.1038/s41598-020-73369-6

**Published:** 2020-10-05

**Authors:** Li Jin, Shi-Yang Qin, Rui Zhang, Meng-Wei Li

**Affiliations:** 1 School of Instrument and Electronics, North University of China, Taiyuan, 030051 China; 2 Nantong Institute of Intelligent Opto-Mechatronics, North University of China, Nantong, 226000 China; 3 Academy for Advanced Interdisciplinary Research, North University of China, Taiyuan, 030051 China; 4 Key Laboratory of Instrumentation Science & Dynamic Measurement, Ministry of Education, North University of China, Taiyuan, 030051 China

**Keywords:** Design, synthesis and processing, Techniques and instrumentation

## Abstract

Micro-electro-mechanical system (MEMS) gyroscopes have numerous potential applications including guidance, robotics, tactical-grade navigation, and automotive applications fields. The methods with ability of the weak Coriolis force detection are critical for MEMS gyroscopes. In this paper, we presented a design of MEMS gyroscope based on the tunneling magneto-resistance effect with higher detection sensitivity. Of all these designed parameters, the structural, magnetic field, and magneto-resistance sensitivity values reach to 21.6 nm/°/s, 0.0023 Oe/nm, and 29.5 mV/Oe, thus, with total sensitivity of 1.47 mV/°/s. Multi-bridge circuit method is employed to suppress external magnetic interference and avoid the integration error of the TMR devices effectively. The proposed tunneling magneto-resistive micro-gyroscope shows a possibility to make an inertial grade MEMS gyroscope in the future.

## Introduction

With the rapid development of MEMS technology, MEMS gyroscopes have been widely used in various applications, including consumer electronics, automobiles, industrial control systems and inertial navigation applications^1–4^. These applications urgently require MEMS gyroscopes with higher sensitivity, lower noise and larger bandwidth^5–9^. To improve the performance of the MEMS gyroscopes, lots of researches have been done on the weak Coriolis force detection method of the MEMS gyroscopes. Most of the MEMS gyroscopes have been realized by the capacitance^10–13^, piezoelectric^2,14–16^ and piezo-resistive methods^17,18^. The capacitance detection method is vulnerable to electromagnetic interference^10^, and limited detection sensitivity has been the major issue to enhance the sensitivity of MEMS gyroscopes. Due to the bias stability and correction speed, the piezoelectric method is not suitable for continuous testing^14,15^. Moreover, it is difficult to improve the sensitivity of piezo-resistive-based MEMS gyroscopes because of their inherent temperature effect^17^.

However, the pursuit of MEMS gyroscopes based on new physical effects has never stopped^19–24^. An all-integrated nano-photonic optical gyroscope based on Sagnac effect is demonstrated to reduce thermal fluctuations and mismatch by exploiting the reciprocity of passive optical networks, increasing its sensitivity significantly^19^. A kind of MEMS piezoelectric solid disk gyroscope is proposed, which operates in an in-plane elliptic bulk acoustic wave (BAW) mode, and has an angular rate sensitivity of about 340 μV/°/s and remains linear with applied rotation rate as high as 500°/s^20^. In addition, a novel resonant square gyroscope based on a group of orthogonal degenerate modes can be efficiently transduced and operated using thin piezoelectric films, which show a linear rate sensitivity of 20.38 µV/°/s^21^. Moreover, an optical micro-gyroscope based on nano-grating detection enables high sensitivity detection with 3.03 mV/°/s, and represents a significant improvement in the detection precision of the micro-gyroscope^22^. Further investigations, such as micro-atomic gyroscope (MAG)^23,24^ and micro-fluid gyroscope (MFG)^25^, will also improve the performance of the micro-gyroscopes. The comparison of the characteristics and performance of the gyroscopes based on different mechanisms is summarized in Table 1.

**Table 1:** Comparison of the characteristics and performance of the gyroscopes based on different mechanisms.

Gyroscope type	Characteristics	Performance
Capacitance^10–13^	Compatible with integrated circuits Vulnerable to electromagnetic interference	Sensitivity: 0.2 mV/º/s^12^ Noise floor: 0.01 º/s/√Hz^12^
Piezoelectric^2,14–16^	Good robustness, wide measuring range, and high resistance to shock Work in atmospheric environment, need correction to compensate the temperature-induced drift	Sensitivity: 4.5 mV/º/s^2^ Angle random walk: 0.05°/√h
Piezoresistive^17,18^	Detection accuracy limited by temperature effect	Sensitivity: 1.48 Ω/rad/s^18^ Noise floor: mdps/√Hz^18^
Fibre-optic (Sagnac effect)^19^	High precision and widely dynamic range Long lifetime, robustness to the environment and high cost	Sensitivity: 0.25 μV/°/s Bias instability: 1 r.p.m Angle random walk: 650°/√h
BAW^2,20^	High resonant frequency and Q-factor Low noise, high linearity, sustaining electronics noise and high bandwidth	Sensitivity: 0.32 mV/º/s^2^ Noise floor: 0.37º/√h/√Hz^2^
Nano-grating^22^	Detection with low noise and high sensitivity Immunity to electromagnetic interference and pull-down effect	Sensitivity: 3.03 mV/º/s Noise floor: 5.95 × 10^−5^ º/s/√Hz
MAG^23,24^	Potential high sensitivity and low power consumption High requirement for measurement and detection	Sensitivity: 7 × 10^−5^ º/s/√Hz^24^ Angle random walk: 4.2 × 10^−3^°/√h^24^
MFG^2,25^	Good robustness and ultra-low cost With low precision	Sensitivity: 0.4 mV/°/s^25^
TMR (this work)	High sensitivity, low noise floor and large operation range Suppression of external magnetic interference	Sensitivity: 1.47 mV/°/s Noise floor: 6.8 × 10^−5^ º/s/√Hz

In this letter, we designed a kind of in-plane MEMS gyroscope based on the tunneling magneto-resistance (TMR) effect. TMR is a magneto-resistive effect that occurs in a magnetic tunnel junction^26–29^, which is a component consisting of two ferromagnets separated by a thin insulator. If the insulating layer is thin enough (typically a few nanometers), electrons can tunnel from one ferromagnet into the other, thus changing the resistance of magnetic materials. The TMR device, as the critical component of gyroscope, has high sensitivity to the weak variation of magnetic field. Based on the characteristics of the TMR device, TMR gyroscope gave a total sensitivity of 1.47 mV/º/s along with the noise floor of 6.8 × 10^–5^ º/s/Hz^1/2^, which is comparable to the other mechanism gyroscopes. In addition, we also applied a multi-bridge circuit method to suppress external electromagnetic interference and avoid the integration error of the TMR devices, thus enhancing the dynamic operation accuracy of TMR gyroscope.

## Gyroscope design and fabrication

### Design

**Figure 1:**
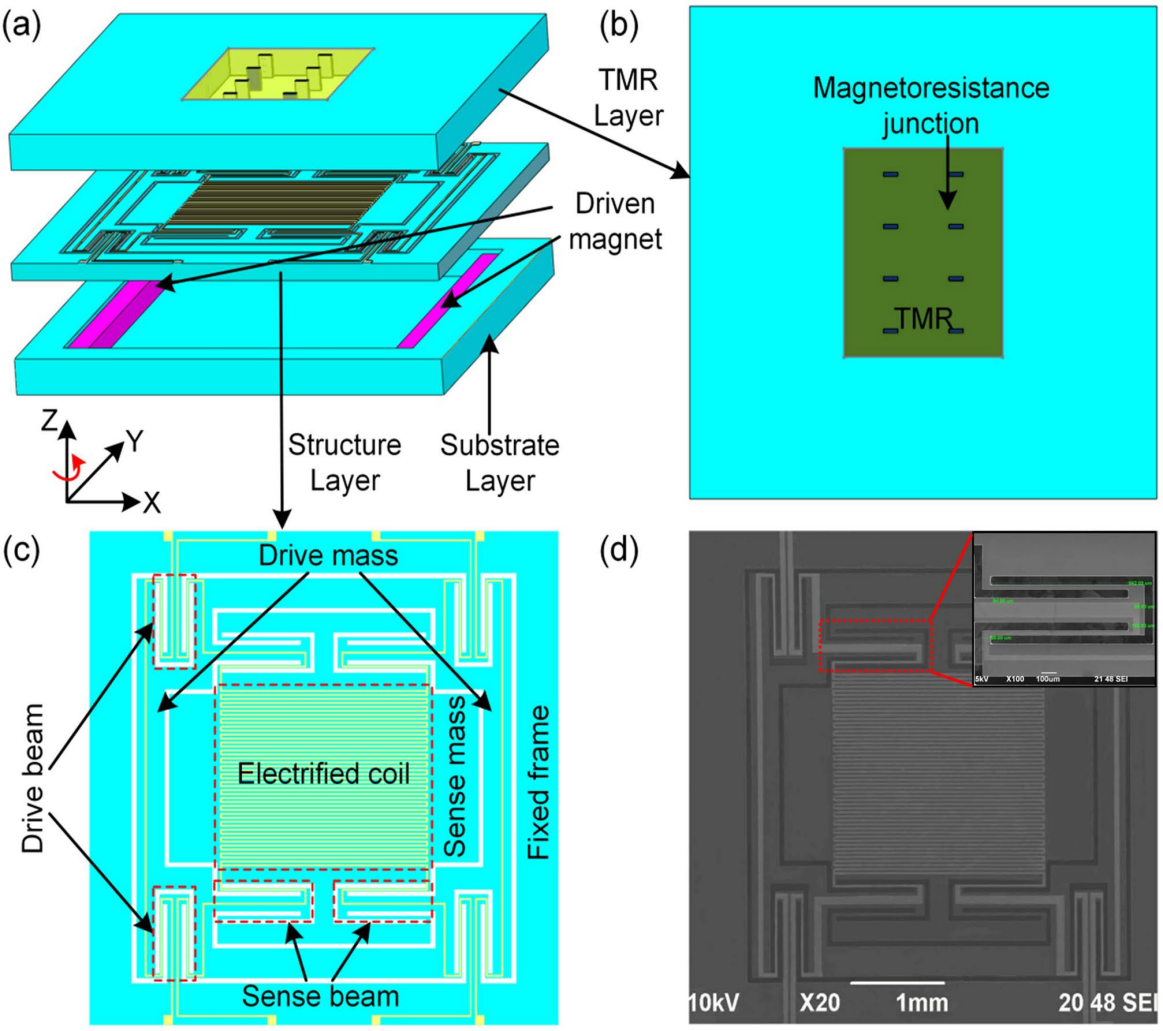
Schematic diagram of the TMR micro-gyroscope. (**a**) The integrated structure of the micro-gyroscope, including the (**b**) TMR layer, (**c**) structure layer and the substrate layer. (**d**) SEM image of the TMR micro-gyroscope. The inset shows the zoomed-in view of the sense beam structure.

The micro-gyroscope structure based on TMR effect consists of three layers, as shown in Fig. 1. The top layer is a TMR structure with several magneto-resistance junctions; the middle layer is the structure layer of the micro-gyroscope, the copper electronic coils are located on the surface of the mass block of the structure; and the bottom layer is the bonding substrate, the two driven magnet are located on the both sides of the substrate, which fabricated from Nd_2_Fe_14_B target sputtered by using pulsed laser deposition, providing a uniform magnetic field in the driving direction. When a voltage is applied to the electrodes of the driven wires, the structure of the micro-gyroscope (including the drive and sense mass block) is driven by the electromagnetic force, thus reciprocating in the X-axis direction. If an angular velocity signal applied on the Z-axis, the sense mass block along with electrified coils will move along the Y-axis direction because of the Coriolis force. The electrified coils provide a uniform magnetic field in the driving direction and highly gradient magnetic field in the sensing direction. This movement will change the magnitude of the magnetic field sensed by the TMR device. Due to the high sensitivity of the TMR device to the weak variation of magnetic field, the resistance value of TMR device changes rapidly when the magnetic field changes. Therefore, the input angular velocity *Ω* of the micro-gyroscope can be obtained by measuring the variation of the output voltage of the TMR device.

The structure of the designed micro-gyroscope includes the drive mass, the sense mass, the electronic coils and the fixed frame, as shown in Fig. 1c. In the driving mode, the drive and sense mass block move along the X-axis direction together. In the sensing mode, the sense mass block moves along the Y-axis direction. The structural sensitivity is defined as the ratio of the displacement variation in sensing direction caused by weak Coriolis force to the change of the input angular velocity *Ω*. The structural sensitivity of micro-gyroscope is susceptible influenced by the frequency difference between the driving mode and the sensing mode. The driving and sensing modes of the micro-gyroscope are analyzed by modal analysis of the Ansys 19.2. According to the desired performance criteria^30,31^, the optimized structural parameters of micro-gyroscope are shown in Table 2.

**Table 2:** The structural parameters of the micro-gyroscope.

Parameters	Value	Parameters	Value
Density	2.33 × 10^3^ kg/m^3^	Young’s modulus	130 GPa
Poisson’s ratio	0.3	Structural thickness	100 µm
Drive beam length	700.5 µm	Sense beam length	842 µm
Drive beam width	30 µm	Sense beam width	30 µm
Drive beam stiffness	7978.6 N/m	Sense beam stiffness	4600.8 N/m
Drive mass	2.8308 × 10^–6^ kg	Sense mass	1.7597 × 10^–6^ kg

### Fabrication process

**Figure 2:**
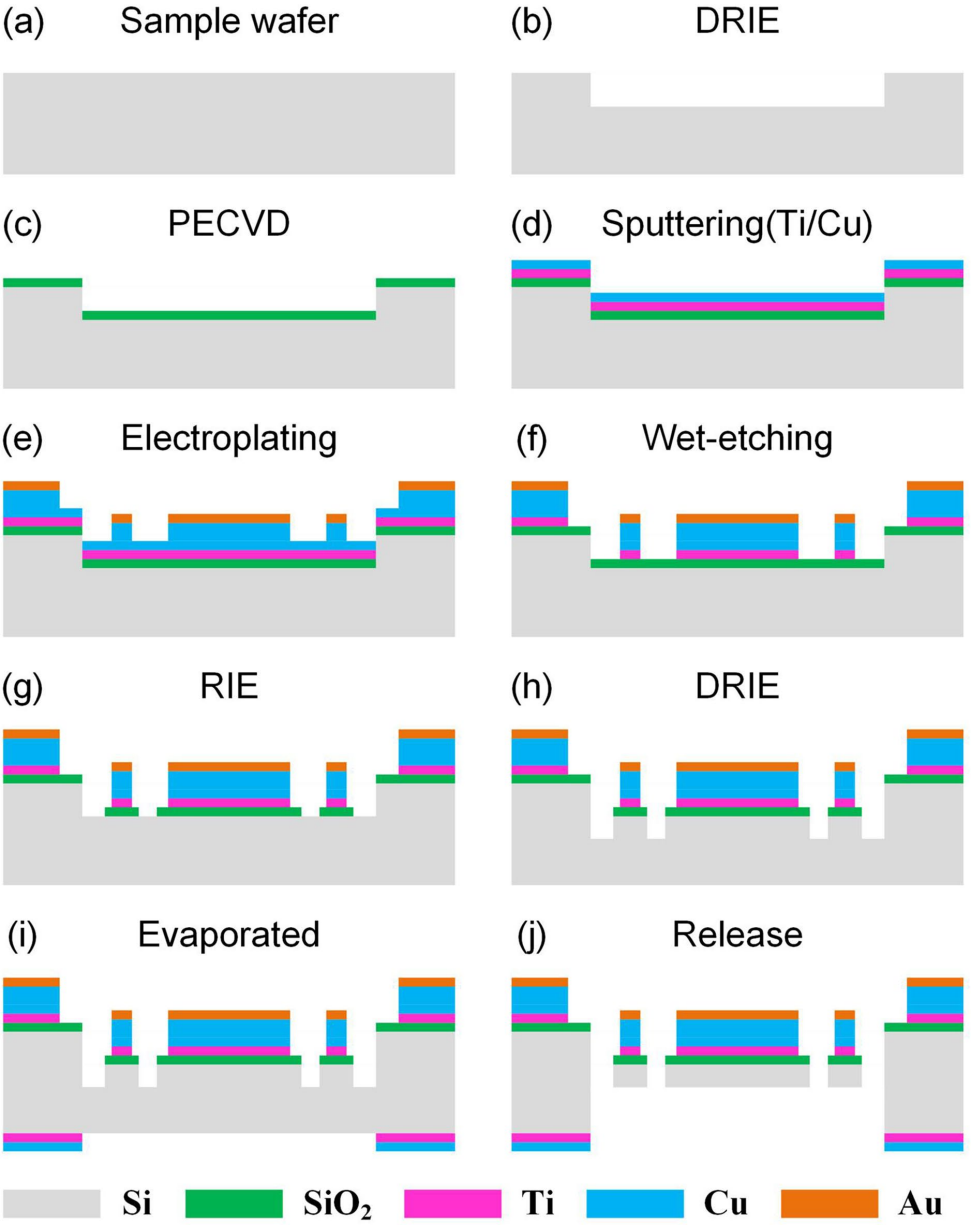
Fabrication process flow of the micro-gyroscope structure.

The gyroscopes are fabricated with silicon-on-insulator (SOI)-wafer-based micromachining process^32^, where a simplified flow of the fabrication is shown in Fig. 2. The silicon wafer was initially etched through deep-reactive ion etching (DRIE) step for an etch depth of 5 μm (Fig. 2b); Then silicon oxide film 200 nm in thickness was formed on the surface of the silicon wafer through plasma enhanced chemical vapour deposition (PECVD) (Fig. 2c), a 50 nm thick Ti adhesive layer and 100 nm thick Cu seed layer are sputtered on the silicon dioxide (Fig. 2d); Multilayered-metal (Cu/Au) electroplating has been employed to form conductive/protective electronic coils (Fig. 2e), and then etched by the wet method (Fig. 2f); Next, the exposed silicon oxide film was etched by reactive ion etching (RIE) (Fig. 2g), while the silicon layer in diaphragm area was etched by DRIE (Fig. 2h); Finally, a 25 nm thickness of Ti layer and 1.5 μm thickness of Cu layer were then evaporated onto the other side of wafer to form bonding pads (Fig. 2i), the micro-gyroscope structure is released by DRIE etching (Fig. 2j). After MEMS fabrication process, wafer is cut into a 4-mm-wide square by using laser cutting. An SEM image of the fabricated structure of the micro-gyroscope is depicted in Fig. 1d. The inset shows the zoomed-in view of the sense beam structure.

## Results and analysis

**Figure 3:**
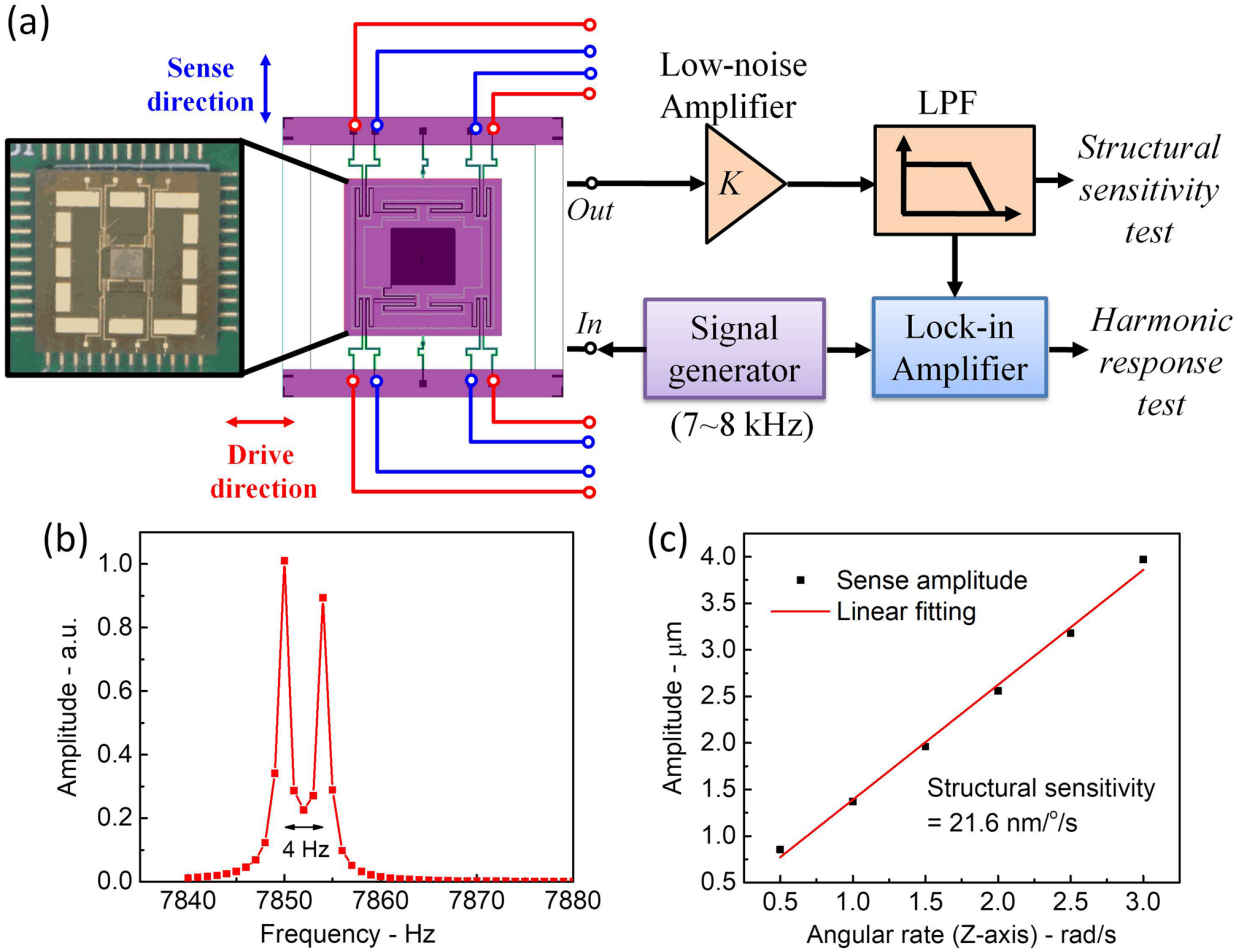
(**a**) Experimental setup for the performance test of the micro-gyroscope. (**b**) The harmonic response result of the TMR micro-gyroscope. (**c**) The dependence of the displacement in the sensing direction on the angular velocity along the *Z*-axis direction. The slope of the linear fitting shows the structural sensitivity of the TMR micro-gyroscope.

It is well-known that the device performance is dependent on micro-fabrication tolerances. With the condition of the current fabrication system we employed, we implement harmonic response test of the micro-gyroscope. Block diagram of experimental setup is shown in Fig. 3a. Harmonic response testing is employed to investigate the mode-matching of the micro-gyroscope. The sweeping frequency AC signal generated by the signal generator used to drive the micro-gyroscope, the output signal of the driving/sensing direction is amplified and low-pass filtered, and then synchronously demodulated by using the lock-in amplifier (AMETEK Model 7270 DSP lock-in amplifier). The result of harmonic response test is shown in Fig. 3b. The resonant frequencies of the driving and sensing modes are tested to be 7850 Hz and 7854 Hz. This 4 Hz frequency split is mainly caused by the asymmetry in the fabrication processing.

Based on the dynamic equations of micro-gyroscope, the structural sensitivity of TMR gyroscope can be expressed as:

1 $$S_{{{\text{str}}}} = \frac{{B_{{\text{y}}} }}{\Omega } = \frac{{2F_{0} \omega }}{{m_{x} \omega_{x}^{2} \omega_{y}^{2} }}\frac{1}{{\sqrt {(1 - (\frac{\omega }{{\omega_{x} }})^{2} )^{2} + \frac{1}{{Q_{x}^{2} }}(\frac{\omega }{{\omega_{x} }})^{2} } }}\frac{1}{{\sqrt {(1 - (\frac{\omega }{{\omega_{y} }})^{2} )^{2} + \frac{1}{{Q_{y}^{2} }}(\frac{\omega }{{\omega_{y} }})^{2} } }}$$

where *Q*_*x*_, *Q*_*y*_ ${\mathrm{Q}}_{\mathrm{x}}, {\mathrm{Q}}_{\mathrm{y}}$ are the quality factor of driving and sensing mode, *ω*_*x*_, *ω*_*y*_ are the natural frequency of driving and sensing mode, respectively. Using Eq. (1), the structural sensitivity is calculated to be 28.8 nm/º/s. On the other hand, based on the micro-gyroscope model and performance characteristics, we study the dependence of the displacements of the sensing direction on the angular velocity along the Z-axis direction with the initial electromagnetic driven force of 400 μN in the X-axis direction. With the result shown in Fig. 3c after linearly fitted, the structural sensitivity of the micro-gyroscope is:

2 $$S_{{{\text{str}}}} = \frac{{B_{{\text{y}}} }}{\Omega } = 21.6\;{\text{nm/}}^\circ {\text{/s}}$$

which agreed with the theoretical value of structural sensitivity.

During the operation of the micro-gyroscope, the magnetic field sensitivity is defined as the ratio between the variation of the magnetic field and the range of displacement of micro-gyroscope along the sense direction. The TMR device requires not only high-sensitivity magnetic field in the sensing direction, but also to avoid the interference of the magnetic field in the driving direction. For these reasons, the magnetic field need to be able to maintain a uniform magnetic field of the same height in the driving direction and has a rapidly change in the sensing direction.

**Figure 4:**
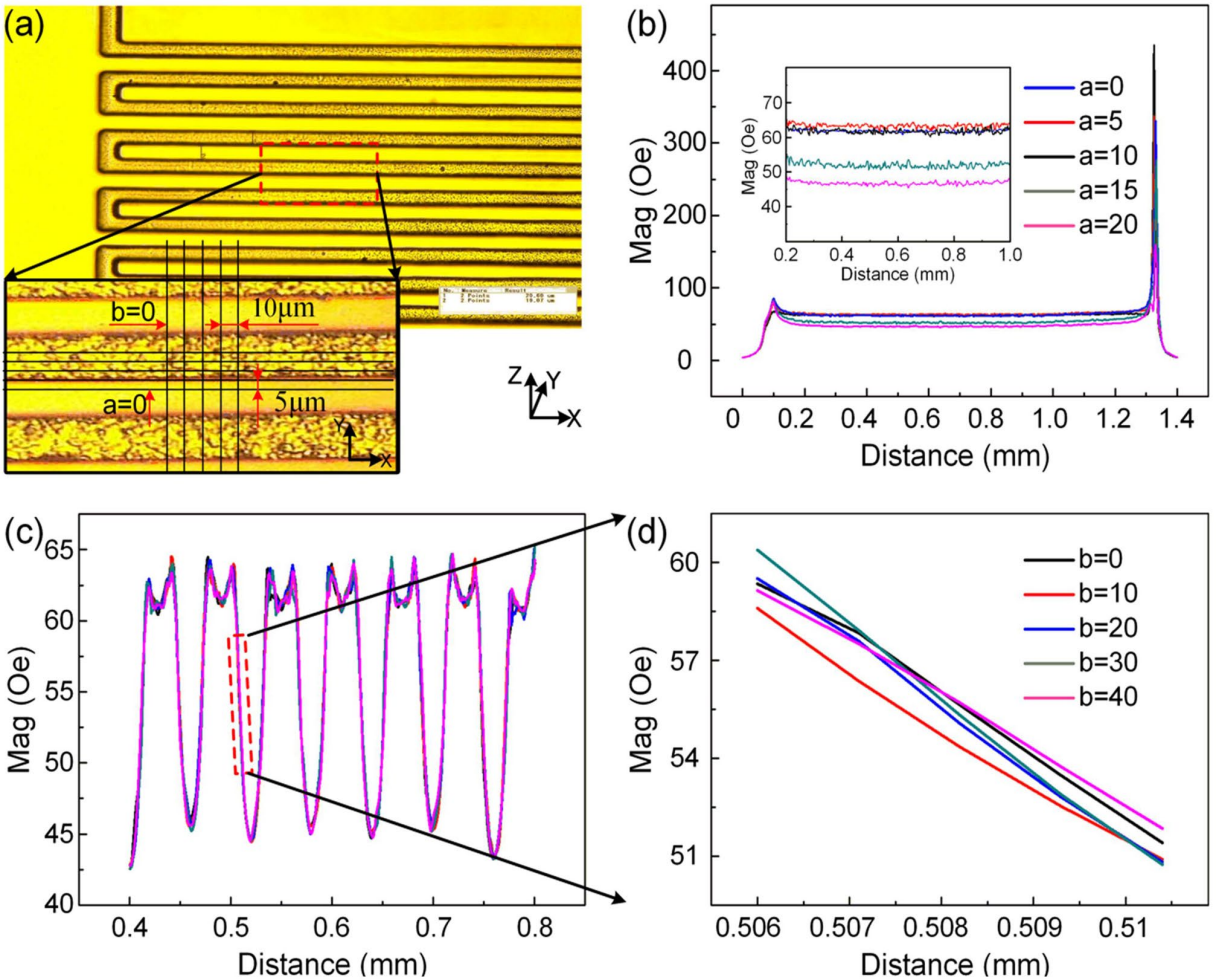
Magnetic field distributions of the electronic coils. (**a**) The position of the detection along the X-axis and the Y-axis, (**b**,**c**) Magnetic field distributions for the driving direction (X-axis)/sensing direction (Y-axis), (**d**) a zoom-in view of (**c**).

To further verify the feasibility of the design, a model of magnetic field distributions of the electronic coils is simulated by Ansoft Maxwell 13.0. From simulations and analyses of the magnetic field sensitivity of TMR gyroscope, we obtain the optimal dimensional parameters with the width of 30 μm, the thickness of 15 μm, and the distance of 30 μm between the adjacent coils. The spatial distribution of the magnetic field can be measured by employing the gauss-meter with resolution of 0.1 mT, we mainly focus on five lines with parallel spacing of 5 μm along the X-axis and spacing of 10 μm along the Y-axis at a height of 1 μm above the electronic coils, as shown in Fig. 4a. The spatial distribution of the magnetic field in the driving direction and sensing direction are shown in Fig. 4b,c, respectively. The results indicate that the magnitude of the magnetic field remains a constant along the driving direction (X-axis), while presents a quasi-sinusoidal distribution of magnetic field in the sensing direction (Y-axis), with a rapid change of magnetic field and good repeatability in the linear range (Fig. 4d). Therefore, the magnetic field sensitivity is written as:

3 $$S_{{{\text{mag}}}} = \frac{\Delta B}{{\Delta d}} = 0.0023{\text{ Oe/nm}}$$

**Figure 5:**
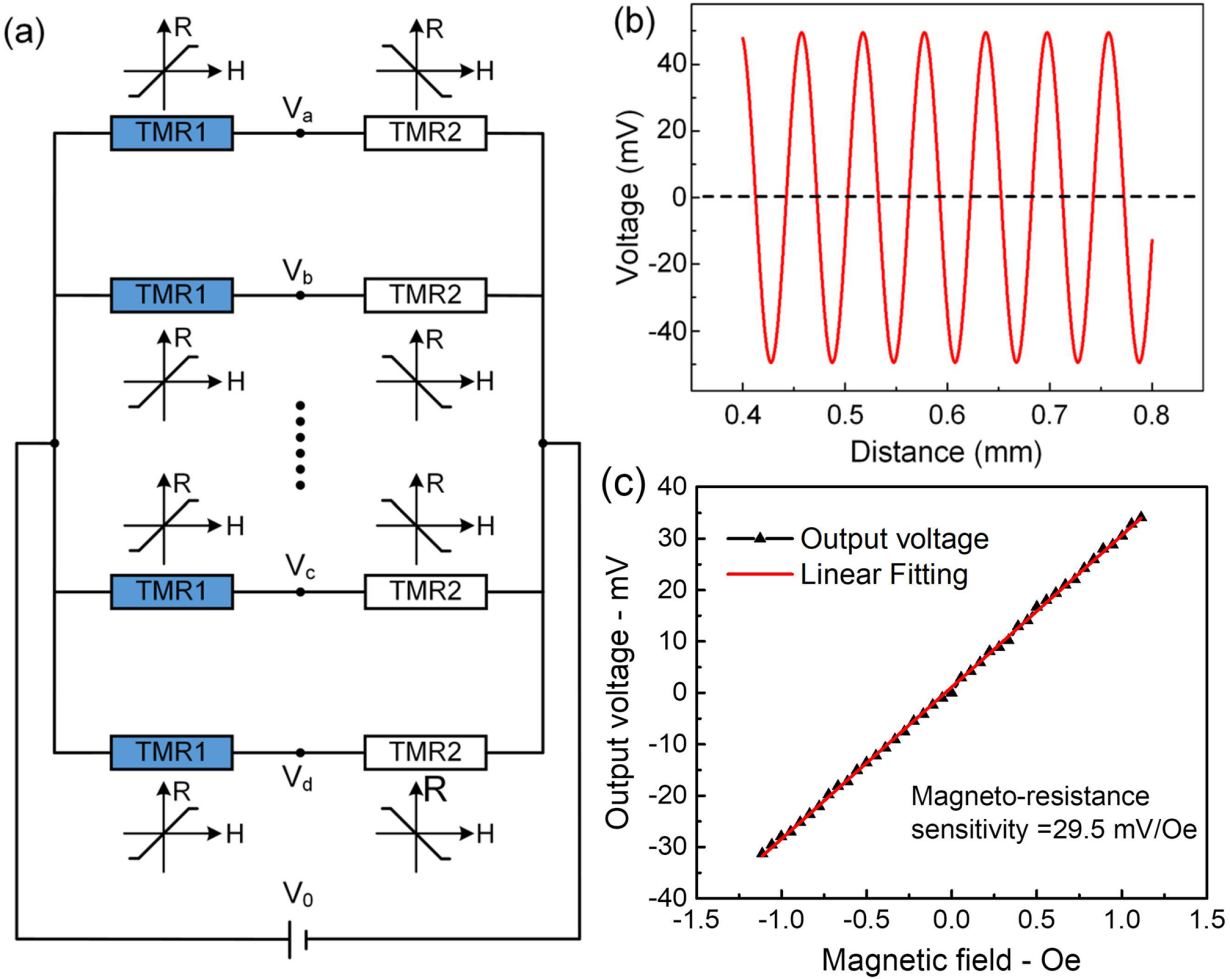
Multi-bridge circuit scheme and testing results of the TMR device. (**a**) Schematic of multi-bridge circuits in TMR micro-gyroscope, (**b**) The baseline offset of differential voltage output is near zero by employing multi-bridge circuit scheme, (**c**) Experimental results of the magneto-resistance sensitivity, the red line represents the linear fit to the data points.

To ensure the measurement sensitivity in TMR device operation, it’s necessary to minimize the influence of external magnetic field on the performance of the TMR device and then increase their detection stability. We proposed the multi-bridge circuit scheme in the TMR micro-gyroscope, which is promising method for suppression of the external disturbance influence, as shown in Fig. 5a.

The distribution of the magnetic field is a quasi-cosine function curve in the sense direction. The magnetic field sensed by TMR in each bridge circuit can be expressed as:

4 $${B_i} = {B_0} + A \cdot \cos \left( {2\uppi \frac{{x + \left( {i - 1} \right)d}}{{2D}}} \right)$$

where *B*_0_ is the static magnetic field, *A* is the amplitude of magnetic field, *D* is the line spacing of electric coils, and *d* is the spacing distance of the adjacent bridge circuits.

The TMR layer of the designed micro-gyroscope consists of two kinds of magneto-resistance junctions with opposite performance (TMR_1_ and TMR_2_). The resistance of the TMR_1_ is positively correlated with the magnetic field, whereas the resistance of the TMR_2_ is negatively correlated with the magnetic field. Thus, the resistance value of the TMR_1_ and the TMR_2_ in the corresponding bridge circuits can be written as:

5 $$R_{ij} = R_{0} + ( - 1)^{j} \cdot K \cdot B_{i}$$

where *i* (*i* = 1–4) is the number of the bridge, *j* (*j* = *1,2*) is the TMR type of the bridge circuit, *K* is the sensitivity of the TMR device.

Combined the Eqs. (4) with (5), two voltage output signals for the TMR device (Fig. 5a) are obtained as follows:

6 $$V_{a} = V_{{0}} \cdot \frac{{R_{11} }}{{R_{11} + R_{12} }}$$

7 $$V_{b} = V_{{0}} \cdot \frac{{R_{21} }}{{R_{21} + R_{22} }}$$

Therefore, the differential voltage output of any two adjacent bridges in the multi-bridge circuits is:

8 $$V{ = - }\frac{{V_{{0}} KA}}{{R_{{0}} }}\sin \frac{{{\pi (}2x{ + }d{)}}}{{{2}D}} \cdot \sin \frac{{{\uppi }d}}{{{2}D}}$$

Note that in Eq. (8) the output signal does not depend on the external magnetic field *B*_0_, hence the multi-bridge method can be used to suppress the influence of the external magnetic interference and also avoid the integration error of the TMR device. Furthermore, it can avoid the decrease of measurement accuracy due to the instability of external magnetic field. Figure 5b shows the differential voltage output as a function of the distance, note that the baseline offset of differential voltage output suppress to zero in multi-bridge circuit scheme.

The experimental test results of the TMR device is shown in Fig. 5c. Magneto-resistance sensitivity is defined as the ratio of voltage variation of the TMR device to the change in the spatial magnetic field. According to the test data and linear fit, the magneto-resistance sensitivity of the TMR device is obtained:

9 $$S_{{{\text{TMR}}}} = \frac{\Delta V}{{\Delta B}} = 29.5{\text{ mV/Oe}}$$

The total sensitivity of the designed TMR micro-gyroscope consists of the structural sensitivity, magnetic field sensitivity and magneto-resistance sensitivity. Based on the above experimental results and analysis, we obtain sensitivity values of 21.6 nm/°/s, 0.0023 Oe/nm, and 29.5 mV/Oe, respectively. Therefore, the total sensitivity of the TMR micro-gyroscope is:

10 $$S = \, S_{str} \cdot S_{mag} \cdot S_{{{\text{TMR}}}} = 1.47\;{\text{mV}}/^\circ /{\text{s}}$$

To enhance the overall sensitivity of TMR gyroscope, we employed the magneto-resistance device with higher sensitivity and guaranteed its operation in the linear region simultaneously. However, the magneto-resistance device has higher sensitivity to the weak variation of magnetic field at the expense of its linear operation range. Therefore, the measurement range of TMR gyroscope is mainly limited by the operation range of the magneto-resistance device. According to the relationship between the output of the magneto-resistance device and the magnitude of the detected magnetic field, the operation range is approximately ± 30 Oe, corresponding to the dynamic range of ± 600°/s.

To investigate the various noises which establish the sensitivity limitations of TMR gyroscope, we analyzed the noise floor of TMR gyroscope and identified its scaling limits. Brownian motion of the proof mass caused by molecular collisions from the surrounding environment represents the fundamental limiting noise component of TMR gyroscope^30^. The Brownian thermo-mechanical noise is given by:

11 $${\Omega _B} = \frac{1}{{{B_x}{\omega _x}}}\sqrt {\frac{{{k_B}T{\omega _y}}}{{{m_y}{Q_y}}}}$$

where *k*_*B*_ is the Boltzmann’s constant, *T* is the ambient temperature, *ω*_*x*_(*ω*_*y*_) is the resonant frequency of the driving mode (sensing mode), *B*_*x*_ is the driven displacement, *m*_*y*_ is the sense mass, and *Q*_*y*_ is the quality factor of the sense direction. According to the expression of Brownian thermo-mechanical noise, it can be seen that the performance of the micro-gyroscope can be improved by increasing the amplitude of the driving mode or with high quality factor. In addition, the electronic noise of the TMR micro-gyroscope mainly originates from *1/f* noise of laser relative intensity noise (*RIN*) and the amplifier noise. The electronic noise of the TMR micro-gyroscope can be written as: (where the noise spectral density (*PSD*) of the TMR device in the 10^–7^ V/Hz level).

12 $${\Omega _e} = \frac{{PSD}}{S}$$

Since the Brownian and the electronic noise are uncorrelated, the overall noise floor of the TMR micro-gyroscope is then given by^12^:

13 $${\Omega _{total}} = \sqrt {\Omega _B^2 + \Omega _e^2}$$

Thus, the low-noise characteristics of the TMR micro-gyroscope can be realized with the overall noise floor of 6.8 × 10^–5^ º/s/Hz^1/2^, which are superior to some current type of micro-gyroscopes.

## Conclusion

This paper presents the design, fabrication and testing of a high aspect-ratio 100 μm thick, 4 mm wide in-plane TMR micro-gyroscope, which enables the detection of the weak Coriolis force of the micro-gyroscope. A model for the total sensitivity of TMR micro-gyroscope is established that indicates the designed structure, magnetic field together with magneto-resistance devices give important contributions. Most important of all, the measurement accuracy depends on the external magnetic field fluctuations. Thus, the multi-bridge circuit method is proposed to suppress external magnetic interference and avoid the integration error of the TMR devices effectively, enhancing the accuracy of operation. Combined the TMR effect with multi-bridge circuit method, the total sensitivity of TMR gyroscope is 1.47 mV/°/s, and the noise floor is 6.8 × 10^–5^ º/s/Hz^1/2^. The TMR micro-gyroscope offering good performance will broaden the range of application, which lays a foundation for obtaining the inertial devices with higher sensitivity in the future.
